# Impact of the Pd_2_Spm (Spermine) Complex on the Metabolism of Triple-Negative Breast Cancer Tumors of a Xenograft Mouse Model

**DOI:** 10.3390/ijms221910775

**Published:** 2021-10-05

**Authors:** Tatiana J. Carneiro, Rita Araújo, Martin Vojtek, Salomé Gonçalves-Monteiro, Ana L. M. Batista de Carvalho, Maria Paula M. Marques, Carmen Diniz, Ana M. Gil

**Affiliations:** 1 Department of Chemistry and CICECO-Aveiro Institute of Materials, University of Aveiro, 3810-193 Aveiro, Portugal; tatiana.joao@ua.pt (T.J.C.); anarita.asilva@ua.pt (R.A.); 2 LAQV/REQUIMTE, Department of Drug Sciences, Laboratory of Pharmacology, Faculty of Pharmacy, University of Porto, 4150-755 Porto, Portugal; matovoj@gmail.com (M.V.); salomemonteiro8180@gmail.com (S.G.-M.); 3 “Química-Física Molecular”, Department of Chemistry, University of Coimbra, 3004-535 Coimbra, Portugal; almbc@uc.pt (A.L.M.B.d.C.); pmc@ci.uc.pt (M.P.M.M.); 4 Department of Life Sciences, Faculty of Science and Technology, University of Coimbra, 3000-456 Coimbra, Portugal

**Keywords:** platinum(II), palladium(II), spermine, cisplatin, human triple-negative breast cancer, xenografts, mice, NMR, metabolomics

## Abstract

The interest in palladium(II) compounds as potential new anticancer drugs has increased in recent years, due to their high toxicity and acquired resistance to platinum(II)-derived agents, namely cisplatin. In fact, palladium complexes with biogenic polyamines (e.g., spermine, Pd_2_Spm) have been known to display favorable antineoplastic properties against distinct human breast cancer cell lines. This study describes the in vivo response of triple-negative breast cancer (TNBC) tumors to the Pd_2_Spm complex or to cisplatin (reference drug), compared to tumors in vehicle-treated mice. Both polar and lipophilic extracts of tumors, excised from a MDA-MB-231 cell-derived xenograft mouse model, were characterized through nuclear magnetic resonance (NMR) metabolomics. Interestingly, the results show that polar and lipophilic metabolomes clearly exhibit distinct responses for each drug, with polar metabolites showing a stronger impact of the Pd(II)-complex compared to cisplatin, whereas neither drug was observed to significantly affect tumor lipophilic metabolism. Compared to cisplatin, exposure to Pd_2_Spm triggered a higher number of, and more marked, variations in some amino acids, nucleotides and derivatives, membrane precursors (choline and phosphoethanolamine), dimethylamine, fumarate and guanidine acetate, a signature that may be relatable to the cytotoxicity and/or mechanism of action of the palladium complex. Putative explanatory biochemical hypotheses are advanced on the role of the new Pd_2_Spm complex in TNBC metabolism.

## 1. Introduction

Breast cancer (BC) is one of the most common types of cancers identified worldwide [1], with female BC ranking first for incidence in 159 countries out of 185 [1,2]. Projections for the next 10 to 20 years reveal that BC will account for ca. 3 million (11%) [3] of the estimated 30 million newly diagnosed cancer cases [3,4], contributing importantly to the high rates of cancer mortality [3,5]. BC is a heterogenous disease [6], and it can be classified into different molecular subtypes [7,8] according to the expression of the (i) human epidermal growth factor receptor 2 (HER2), (ii) estrogen and progesterone receptors (ER and PR, respectively), and/or (iii) the cellular proliferation marker Ki-67a. Four main subtypes are defined: luminal A, luminal B, HER2-positive and triple-negative breast cancer (TNBC). The latter is characterized by early incidence (usually before 40 years of age), and is associated with an aggressive phenotype, high metastatic potential and low five-year survival rate (ca. 30%), thus corresponding to poor prognosis [9,10]. These clinical features are explained by the absence of tumor response to hormonal-/antibody-targeted therapies [10], since TNBC does not express any of the ER, PR or HER2 receptors [7,8]. Thus, current treatment options against TNBC rely mostly on chemotherapeutic protocols that involve the administration of anthracyclines, taxanes, antimetabolites and/or alkylating agents, such as platinum (Pt(II))-derived drugs [11,12,13]. Some of these chemotherapeutic agents are, however, often associated with reduced specificity in relation to molecular targets, acquired resistance and high toxicity [14,15,16]. Hence, ongoing efforts are needed to identify new molecular markers for TNBC as possible therapeutic targets, with a view to developing new and more effective drugs [17,18].

Metabolic reprogramming is recognized as an important hallmark of cancer, reflective of the interplay of the tumor with its microenvironment, and much has been endeavored in describing and understanding TNBC metabolic traits [19]. In particular, metabolomic strategies have been extensively used to characterize the TNBC metabolome, mostly through the study of cell lines [20,21,22,23,24,25,26,27,28,29,30,31,32,33,34,35,36,37,38,39,40,41], but also of human samples, either patient biofluids (mainly plasma and serum [42,43,44,45,46,47,48], but also saliva [49]) and tumor/tissue biopsies [44,50,51,52] or extracts [53,54,55]. In vivo animal studies (e.g., xenograft models [56,57,58,59,60]), however, are still scarce, compared to in vitro reports, despite the capacity of an in vivo animal model to represent the complex response of the whole organism, both to the disease and to therapy [61]. In some instances, attempts have been made to correlate in vitro and in vivo metabolic traits of TNBC [62,63]. Reported metabolomic studies have mostly addressed (i) tumor profiling, in the search for biomarkers of diagnosis/prognosis (comparing TNBC tumors with controls [45,48,49] or other BC subtypes [21,22,25,46,52]), and (ii) tumor response to novel treatment protocols [26,28,32,44,60]. In addition, the metabolic adaptations of tumors to specific conditions, such as methionine sensitivity (related to tumor cells proliferation) [38], the expression of the Mucin1 glycoprotein (modulatory role in cancer metabolism) [39], the depletion of membrane protein myoferlin (and subsequent impact on metastasis extension) [63], hypoxia [40,62], the extent of glucose metabolism (associated with tumor malignancy) [41] and breast cancer gene 1 (BRCA1) mutations [42], have been investigated. Some metabolomic studies have focused on the impact of cytotoxic agents, such as bevacizumab, paclitaxel, doxorubicin, tamoxifen, and cisplatin (cDDP), on TNBC metabolism [60], as well as on the possibility of the prediction of treatment efficacy [26,44]. Notably, variations in lactate, acetate, and phosphocholine have been considered to constitute a specific signature distinguishing cDDP responder and non-responder MDA-MB-231 cells [28], in tandem with shifts in arginine and polyamines levels as the response of MDA-MB-468 and SUM-159PT cells (corresponding to basal-A and basal-B TNBC, respectively [64]) to cDDP and doxorubicin [32]. However, adverse effects of cDDP and other Pt(II) drugs (namely, toxicity and acquired resistance) have been reported [16], explaining the continuing search for other drugs, including those containing metal centers [65,66]. Palladium (Pd(II)) complexes have been extensively tested against human TNBC cell lines [67], with promising results from using Pd(II) chelates with biogenic polyamines [68,69], such as spermine (Spm = H_2_N(CH_2_)_3_NH(CH_2_)_4_NH(CH_2_)_3_NH_2_) [70,71,72]. In fact, Pd_2_Spm has antiproliferative properties via disrupting cytoskeletal microtubules, leading to both cell morphology impairment [70] and cell apoptosis [71]. Additionally, the antimetastatic properties of Pd_2_Spm have been demonstrated through its anti-angiogenic and anti-migratory effects [72]. Moreover, Pd_2_Spm has exhibited favorable pharmacokinetics and biodistribution in healthy mice [73], thus increasing the interest in this compound as a promising pharmacological agent for cancer treatment. However, the potential cytotoxic effect of Pd_2_Spm needs to be further validated in in vivo cancer models, and this is the subject of ongoing work in our group.

The present paper reports, for the first time to our knowledge, a metabolic evaluation of the in vivo response of an MDA-MB-231 cell-derived xenograft (CDX) mouse model to Pd_2_Spm, compared to cisplatin, through nuclear magnetic resonance (NMR) metabolomics of polar and lipophilic extracts of the resulting TNBC tumors. Metabolic markers and putative biochemical interpretations are advanced to tentatively explain the relative effects of the two drugs, Pd_2_Spm and cDDP, on tumor metabolism. 

## 2. Results

**Figure 1:**
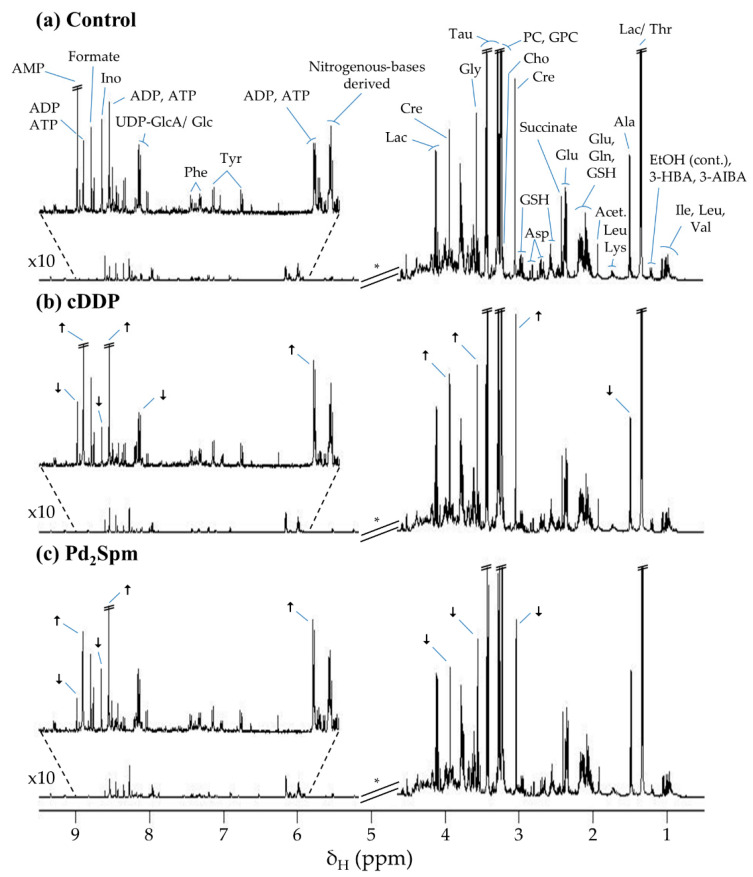
Average 500 MHz ^1^H nuclear magnetic resonance (NMR) spectra of aqueous extracts of tumors from MDA-MB-231 cell-derived xenograft (CDX) mouse model after exposure to (**a**) vehicle (phosphate-buffered saline, PBS), (**b**) cDDP, and (**c**) Pd_2_Spm. * Cut-off of water suppression region (δ 4.60–5.20), not considered in the multivariate analysis. The arrows identify visually apparent metabolic variations between treated groups and controls. Abbreviations: 3-letter code for amino acids; 3-AIBA, 3-aminoisobutyric acid; 3-HBA, 3-hydroxybutyrate; Acet., acetate; ADP, adenosine diphosphate; AMP, adenosine monophosphate; ATP, adenosine triphosphate; Cho, choline; Cre, creatine; EtOH, ethanol (contaminant); GPC, glycerophosphocholine; GSH, glutathione (reduced); Ino, inosine; Lac, lactate; PC, phosphocholine; Tau, taurine; UDP-GlcA/ Glc, uridine diphosphate-glucuronate/glucose.

The ^1^H NMR spectra of the polar extracts of non-treated tumors and those treated either with cDDP or Pd_2_Spm (Figure 1) present information on a wide number of metabolites, ranging from amino acids to choline compounds, sugars, nucleotides, organic acids and a number of other compounds, as listed in Table S1.

**Figure 2:**
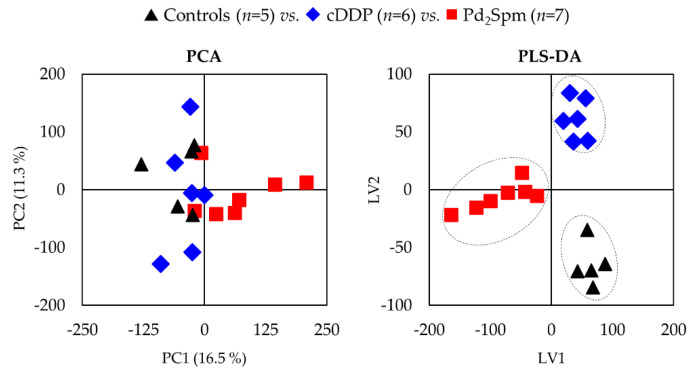
Principal component analysis (PCA) and partial least squares–discriminant analysis (PLS-DA) scores scatter plots for ^1^H NMR spectra of aqueous extracts of tumors from MDA-MB-231 CDX mouse model after exposure to PBS (black triangles, *n* = 5), cDDP (blue diamonds, *n* = 6), and Pd_2_Spm (red squares, *n* = 7).

A principal component analysis (PCA) of the spectra of polar extracts indicates an overlap of controls with cDDP-treated tumors (Figure 2, left), whereas a separation tendency is seen for Pd_2_Spm-treated tumors (Figure 2, left). This suggests that the Pd(II) complex may be exerting a stronger impact on TNBC tumor metabolism. Partial least squares–discriminant analysis (PLS-DA) clearly shows a separation of the three groups (Figure 2, right), which does indicate the distinct effects of the two complexes. 

**Figure 3:**
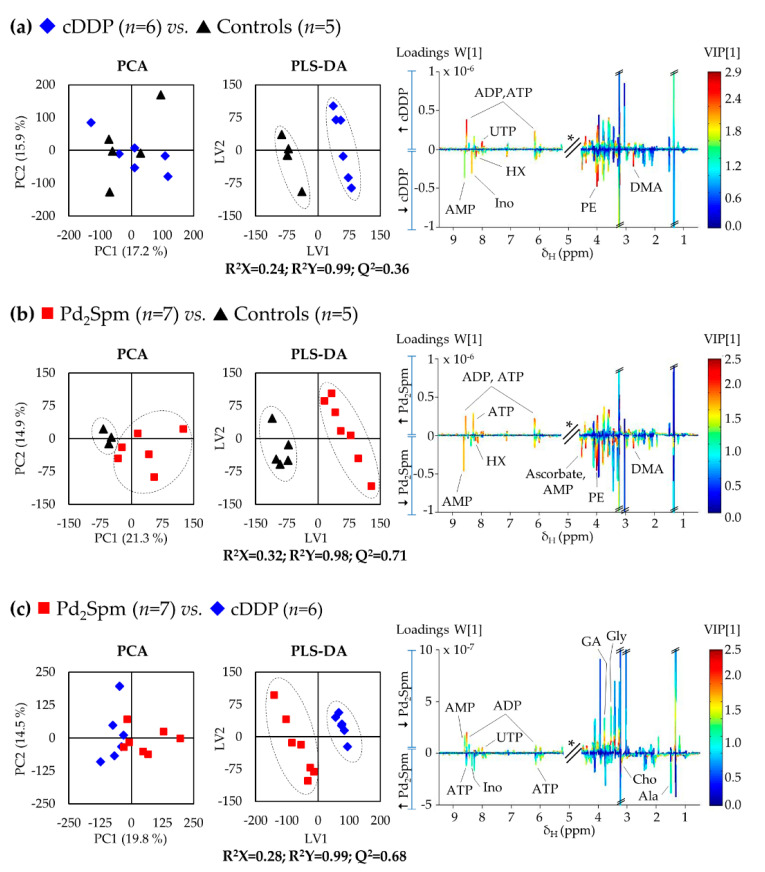
Score scatter plots of PCA (left) and PLS-DA (middle) models obtained for ^1^H NMR spectra of aqueous extracts of tumors from MDA-MB-231 CDX mouse model, obtained for each pairwise analysis: (**a**) cDDP vs. Controls; (**b**) Pd_2_Spm vs. Controls; and (**c**) Pd_2_Spm vs. cDDP. LV1 loadings plots (right) from the correspondent PLS-DA model are colored according to variable importance to projection (VIP) and exhibit the assignment of main peaks (*: cut-off spectral region corresponding to the water resonance). Abbreviations: DMA, dimethylamine; GA, guanidine acetate; HX, hypoxanthine; PE, phosphoethanolamine; UTP, uridine triphosphate. Other abbreviations are defined in the caption of Figure 1.

A systematic pairwise analysis (Figure 3) confirms the weak impact of cDDP on tumor metabolism, as expressed by the low Q^2^ value (0.36) corresponding to the PLS-DA model in Figure 3a. For Pd_2_Spm, treated tumors separate from controls in unsupervised analysis (Figure 3b, left), as expressed by a robust PLS-DA model with good predictive power (Q^2^ = 0.71, Figure 3b, right). Both models may be interpreted in terms of varying metabolite levels, with the aid of the corresponding loadings plots (Figure 3, right). In addition, PCA and PLS-DA carried out for the direct comparison of the two metal complexes identified a robust distinction in the metabolic signatures of the complexes (with a PLS-DA predictive power of Q^2^ = 0.68, Figure 3c). Interestingly, statistical analysis did not reveal any significant changes in the spectra of the lipophilic extracts of the same tumors (Figure S1).

Table 1 lists all statistically relevant metabolite changes between the three pairwise comparisons. It becomes clear that only five metabolites are changed significantly in cDDP-treated tumors, compared to controls, namely: asparagine (inc.), ATP (inc.), hypoxanthine (HX, dec.), uridine triphosphate (UTP, inc.) and dimethylamine (DMA, dec.). On the other hand, Pd_2_Spm induces statistically relevant variations in 10 metabolites, which explains the relatively higher robustness of the corresponding PLS-DA model.

The palladium complex induces changes in asparagine (inc., more marked than with cDDP), choline (inc.), phosphoethanolamine (PE, dec.), fumarate (inc.), ATP (inc., more marked than with cDDP), guanidine acetate (GA, dec.), HX (dec.), DMA (dec., less marked than with cDDP) and unassigned resonances at δ 4.04 (U1, dec.) and δ 8.18 (U2, inc.). All these statistically relevant changes are also noted in Table S1 (arrows in right columns), together with the qualitative tendencies of change (arrows in brackets), and in the heatmap in Figure S2. Furthermore, the direct comparison of the Pd_2_Spm- and cDDP-treated groups (Table 1 and Figure S2, right column) indicates that Pd_2_Spm-treated tumors are also identified by some small metabolite differences (which, however, do not remain statistically relevant when compared to controls), namely, tendencies for raised levels of alanine and the depletion of glycine, and raised levels of NAD^+^, uridine and an unassigned singlet at δ 6.80.

**Figure 4:**
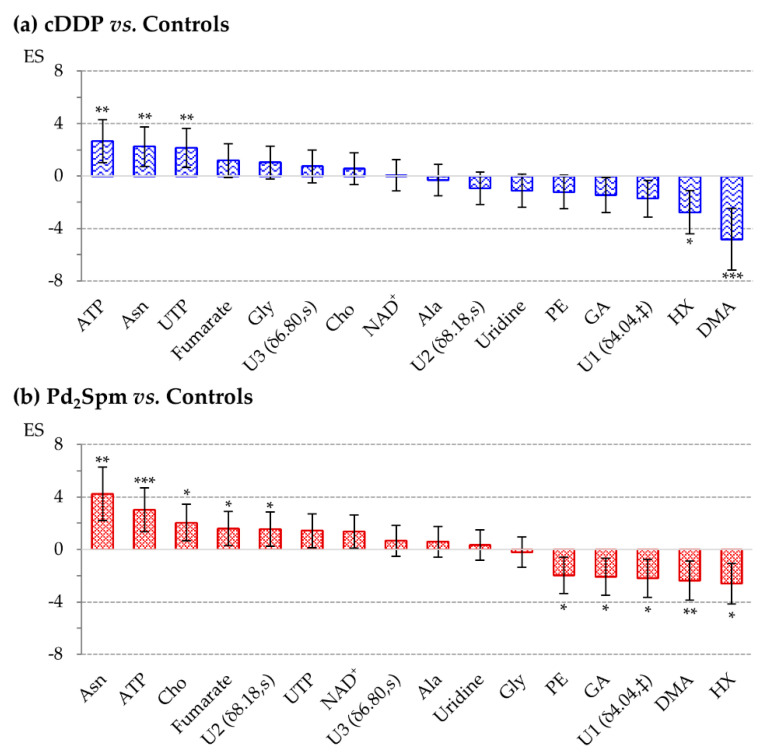
Bar chart illustrating effect size (ES) values [74], from maximum to minimum, for (**a**) cDDP vs. Controls and (**b**) Pd_2_Spm vs. Controls. Error bars represent the error associated to the ES calculation [74]. Asterisks represent the significance level: * *p*-value < 5 × 10^−2^; ** *p*-value < 1 × 10^−2^; *** *p*-value < 1 × 10^−3^ compared to controls. Abbreviations as defined in the captions of Figure 1 and Figure 3, as well as in Table 1; ‡ Partial integration of peak.

The representation of the magnitude and direction of the evolution of the noted metabolite changes (Figure 4) illustrates that, interestingly, each signature comprises the same metabolite players, which, however, differ significantly in magnitude/direction of variation and statistical relevance. Notably, the topmost increased metabolites are in both cases ATP and asparagine, whereas the metabolites showing a larger decrease are (also in both cases) PE, GA, U1, HX and DMA (although in a different order of magnitude).

**Figure 5:**
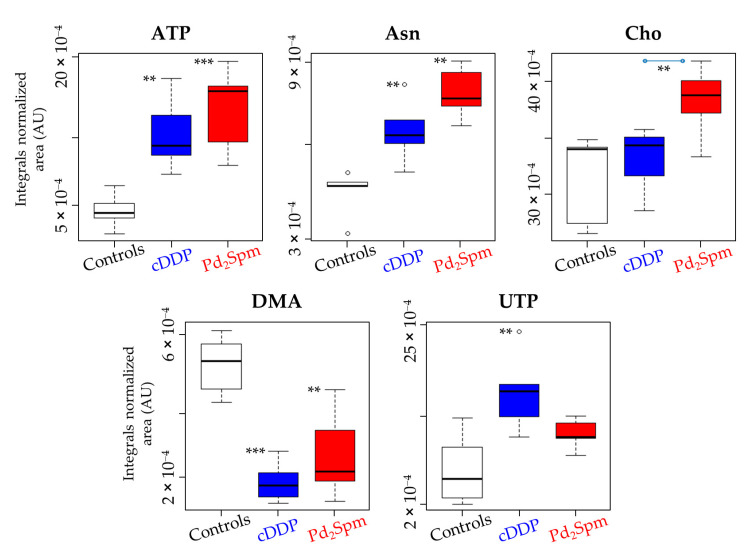
Boxplot representation of metabolites exhibiting variations that remain significant upon false discovery rate correction (^a^ in the Table 1). The box represents the lower and upper quartile (25–75%) with the non-outlier range, the bold line represents the median, and circles (“°”) represent outlier sampls. Asterisks represent the significance level of pairwise comparison with controls (except for Cho, where significance is indicated for drugs comparison, Pd_2_Spm vs. cDDP): ** *p*-value < 1 × 10^−2^; *** *p*-value < 1 × 10^−3^. Abbreviations as defined in captions of Figure 1 and Figure 3.

The features more strongly distinguishing the Pd_2_Spm from the cDDP treatment comprise (i) higher ATP production and lower UTP production, thus not using up uridine; (ii) higher production of choline and NAD^+^; and (iii) lesser depletion of DMA. These distinguishers are clearly illustrated in the boxplots in Figure 5, corresponding to the metabolite changes that survive FDR correction (^a^ in Table 1). 

## 3. Discussion

Firstly, as this is, to our knowledge, the first metabolomics report on cDDP-treated TNBC tumors of a xenograft model, it is relevant to compare these in vivo observations with a previous report of in vitro effects on the same cell line [28]. The human cell line of triple-negative breast cancer MDA-MB-231 was exposed to cDDP (1 µM) and analyzed by ^1^H HRMAS NMR, the effects having been compared to those induced by either doxorubicin or tamoxifen. The following responses were observed: (i) lower levels of lipid-moieties, GA, and acetone (ketone body); (ii) higher protein levels, lactate, acetate, taurine, alanine, glycine, tyrosine, phenylalanine (with slight changes in glutamine and glutamate), and UTP/UDP/UMP. It is interesting to note some similarities between cells and tumor metabolic behavior upon cDDP administration (Figure 4 and Figure S2), namely, a tendency for lower GA and alanine levels (although not significant in tumors) and for higher glycine levels (also not significant in tumors), as well as an increase in UTP. Indeed, an increase in pyrimidine nucleotides has been associated to the DNA damage response induced by the cytotoxic agents cDDP and doxorubicin [32], and the here-detected rise in UTP seems a good indicator of this effect in both cells and tumors. However, in general, less metabolite changes characterize cDDP-treated TNBC tumors, with the metabolic signature reported here lacking changes in lipids, ketone bodies, amino acids (other than asparagine, alanine and glycine), lactate (Warburg effect) and acetate (lipids metabolism). This comparison shows that the in vivo metabolic behavior of MDA-MB-231 cells is attenuated to a large extent, compared to in vitro conditions.

**Figure 6:**
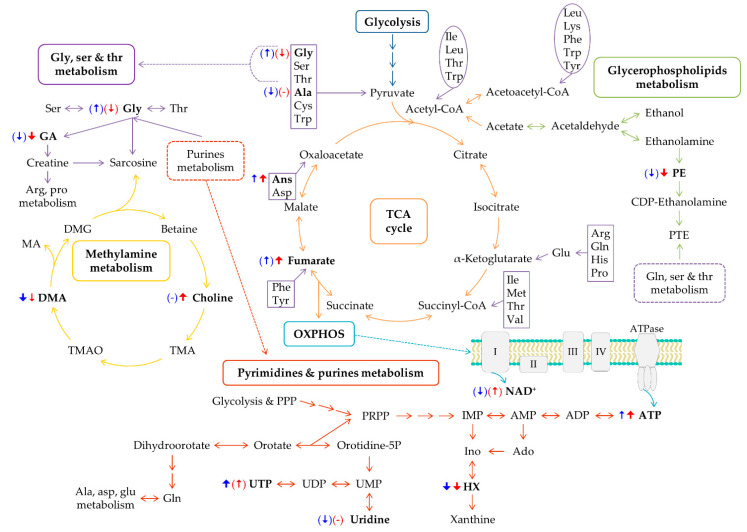
Metabolic pathways putatively identified as the main disturbances on the polar metabolome of TNBC tumors after the exposure to cDDP or Pd_2_Spm complex. The blue and red arrows illustrate the direction of variation, distinguishing each treated mice group, with blue for cDDP and red for Pd_2_Spm, while arrow width is proportional to the statistical significance (ES and *p*-value) of each variation compared to control levels; arrows in brackets represent variations that are not statistical relevant (*p*-value > 0.05). Amino acids involved in anapleurotic reactions and classified as ketogenic and glucogenic are represented in circles and rectangles, respectively. Abbreviations: Ado, adenosine; DMG, dimethylglycine; IMP, inosine monophosphate; MA, methylamine; OXPHOS, oxidative phosphorylation; PPP, pentose phosphate pathway; PRPP, phosphoribosyl diphosphate; PTE, phosphatidylethanolamine; TMA, trimethylamine; TMAO, trimethylamine *N*-oxide; UDP, uridine diphosphate; UMP, uridine monophosphate. Other metabolite abbreviations are defined in the captions of Figure 1 and Figure 3.

Our results show that cDDP-treated tumors (Figure 6, blue arrows) engage three amino acids (asparagine, alanine and glycine), with only asparagine varying with significance (confirming previous reports on extracts of patients TNBC tumors [54]). These amino acids may promote higher TCA activity (consistently with a weak tendency for increased fumarate) for enhanced ATP production. Glycine may also be engaged in serine and threonine metabolism, potentially impacting GA levels. Pyrimidine and purine metabolism is here observed to be affected by the decreased HX and increased UTP (and concomitant uridine decrease), in addition to the marked ATP increase. The use of HX may relate to the active anti-oxidative stress mechanisms, with consistently high HX levels having been reported for non-treated tumors [55]. Finally, the strong DMA depletion induced by cDDP may relate to disturbances in methylamine metabolism, and to choline levels (Figure 6). DMA variations have been observed in several pancreatic and colorectal cancers [76,77], and indeed DMA has been reported to decrease after treatment with epicatechin [78]. It is thus possible that DMA may serve as an indicator of response to cDDP therapy.

Tumor treatment with Pd_2_Spm increases the levels of asparagine, probably to enhance TCA cycle activity, consistently with the more marked fumarate increase, compared to cDDP (Figure 6). Pd_2_Spm-treated tumors are also slightly richer in alanine and more depleted in glycine than cDDP-treated ones (Table 1 and Figure S2) (probably explaining the higher use of GA for creatine/sarcosine synthesis, Figure 6), which supports the distinct interplay of the three amino acids involved in the response to treatment. Reported increases in glycine have often been associated with poor disease prognosis [51], as this metabolite is involved in pathways related to cell proliferation, e.g., the synthesis of proteins, nucleotides and GSH [79,80]. Although no significant changes were noted in glycine compared to controls, the lower levels of glycine in Pd_2_Spm-treated tumors, compared to cDDP-treated tumors, may be suggestive of a better prognosis for the former. The enhanced accumulation of choline seems to suggest a hindrance of choline conversion in the methylamine pathway, leading to less DMA being converted into DMG and subsequently producing sarcosine (Figure 6). This clear deviation in choline/DMA metabolism for Pd_2_Spm-treated tumors may be indicative of a distinct response to the palladium complex. However, a choline enhancement may also be determined by membrane metabolism and, indeed, the new relevance of PE depletion indicates that membrane metabolism is differently affected by the Pd_2_Spm complex. This disturbance can also be expressed by the lower PC/Cho and GPC/Cho ratios (Figure S3), due to the higher choline levels, whereas no significant changes in these ratios are observed upon cDDP treatment. Most changes in choline compounds reportedly related to TNBC have involved alterations in PC and/or GPC levels, which distinguish tumors of different types [56,81] or tumors from non-involved tissue [82]. In turn, the reason for the marked changes in choline alone presently observed (and reflected in the PC/Cho and GPC/Cho ratios) remains unclear at this stage. Finally, in Pd_2_Spm-treated tumors, ATP seems to predominate as an energy source in the decrease in UTP and the precursor uridine, which seem to be required to a larger extent by cDDP-treated tumors (either for energy production and/or for feeding into protein glycosylation processes in the form of glycosylated derivatives).

In future studies, it is important to pursue these issues by searching f or correlations between metabolic characteristics and chemotherapy-induced hepatotoxicity and cardiotoxicity. These are important limiting factors that adversely affect treatment outcomes and are mainly correlated with the accumulation of the chemotherapeutics in these organs [83,84]. Our recent comparative pharmacokinetic study in mice revealed a significantly lower accumulation of palladium (from Pd_2_Spm) in the lungs, brain, liver and heart, compared to platinum (from cDDP) [73]. Therefore, due to its lesser accumulation, Pd_2_Spm is not expected to cause significant deleterious effects (i.e., low cardiotoxicity and hepatotoxicity are expected) compared to cisplatin, thus establishing it as a promising alternative as a putative chemotherapeutic for breast cancer treatment.

## 4. Materials and Methods

### 4.1. Chemicals

Cisplatin (*cis*-dichlorodiammine platinum (II), 99.9%), potassium tetrachloropalladate (II) (K_2_PdCl_4_, 98%) and spermine (*N*,*N*′-bis(3-aminopropyl)-1,4-diaminobutane, 99%) were purchased from Sigma-Aldrich (Sintra, Portugal). Euthasol^®^ solution (400 mg/mL pentobarbital sodium) was obtained from Le Vet (Oudewater, The Netherlands). All reagents were of analytical grade.

The Pd_2_Spm complex was synthesized according to published procedures [85,86]. Briefly, 2 mmol of K_2_PdCl_4_ was dissolved in a small amount of water, and 1 mmol of spermine (in aqueous solution) was added dropwise under stirring. After 24 h, the resulting powder was filtered and washed with acetone (yield 68%). The newly synthesized compound was characterized (and tested for purity) by elemental analysis and vibrational spectroscopy [86].

### 4.2. Ethical Considerations

The handling and care of animals were carried out in full compliance with the Portuguese (Decreto-Lei no. 113/2013) and European (Directive 2010/63/EU) legislation for the protection of animals used for scientific purposes and with the recommendations stated in the Guide for Care and Use of Laboratory Animals of the National Institutes of Health (NIH). The study protocol was approved by the Ethics Committee for Animal Experimentation of the Faculty of Pharmacy of the University of Porto, Porto, Portugal (Permit Number: 25-10-2015), and by the Ethics Committee and the Organ Responsible for the Welfare of Animals of ICBAS-UP, Porto, Portugal (Permit number 134/2015). The Animal Research: Reporting of In Vivo Experiments (ARRIVE) guidelines were followed [87].

### 4.3. Animals Handling Procedures

Female CBA nude mice were acclimatized for 2 weeks at the ICBAS-UP Rodent Animal House Facility (Porto, Portugal). The animals were placed in individually ventilated cages with enrichment material (corncob bedding, paper roll tube, and one large sheet of tissue paper for nesting) and housed in an SPF environment with ad libitum access to water and standard pellet food under controlled 12 h light/dark cycles (lights on at 7.00 AM), temperature (22 ± 2 °C), and humidity (50 ± 10%). At 14 to 17 weeks old, the animals were subcutaneously implanted in left flank with breast cancer MDA-MB-231 cells (25G needle, 5 × 10^6^ cells in 150 µL of PBS). At day 25 post-implantation, when the tumors reached the mean volume of ~250 mm^3^, the mice were randomly allocated into three groups (7 animals per group) using a computer-generated randomization sequence followed by random group allocation to the treatment with either (i) vehicle (phosphate-buffered saline, PBS), (ii) cDDP (2 mg/kg/day), or (iii) Pd_2_Spm (5 mg/kg/day), all administered via intraperitoneal injection (500 µL injection volume) over five consecutive days in the respective group. The animals were monitored for activity, physical condition, determination of body weight, and measurement of tumor growth to guarantee the animals’ welfare. Tumor measurements were performed by two independent researchers using a digital caliper in two perpendicular diameters of the implant, in order to access the experimental conditions and verify the progression of the disease, and as a humane measure. Researchers were blinded to treatment allocation when performing outcome measurements. Two animals from the vehicle group developed ulcerated tumors during the treatment period (day 28 post-implantation), thus these animals were euthanized and excluded from the study. At the day 39 post-implantation (end of the study), animals were euthanized with isoflurane, and the tumors were excised, washed in PBS and weighted (ca. 0.81, 1.07 and 0.90 g for controls (non-treated), cDDP and Pd_2_Spm groups, respectively). The third quartile (bottom, left) of the tumor was selected for the metabolomics analysis. At this point, another animal was excluded from the group exposed to cDDP due to the tumor’s size (0.09 g) being insufficient to allow for all required analyses (metabolomics and other studies). Hence, the final group sizes were as follows: controls *n* = 5; cDDP-treated *n* = 6; Pd_2_Spm-treated *n* = 7.

### 4.4. Tumor Extracts

Frozen tumors were weighted (average weights of 0.038, 0.043 and 0.045 g for controls, cDDP and Pd_2_Spm groups, respectively) and ground to a fine powder by mechanical maceration in liquid N_2_ [88,89,90]. Each sample was recovered into a tube and extracted using the biphasic methanol/chloroform/water (2.0:2.0:1.0) method [91]. Briefly, samples were homogenized in cold 80% methanol (8.0 mL/g), cold 100% chloroform (4.0 mL/g), and cold miliQ water (2 mg/L), vortexed for 60 s and kept at −20 °C for 15 min [91]. Samples were centrifuged (8000 rpm, 5 min, 23 °C), the polar and nonpolar phases were removed into separate vials and vacuum/N_2_ dried, respectively, and stored at −80 °C until further analysis. Before NMR acquisition, aqueous extracts were suspended in 650 µL of 100 mM sodium phosphate buffer (pH 7.4, in D_2_O containing 0.25% 3-(trimethylsilyl)-propionic-2,2,3,3-d4 acid (TSP) for chemical shift referencing), and lipophilic extracts were suspended in 650 µL of CDCl_3_, containing 0.03% tetramethylsilane (TMS). Samples were homogenized and 600 µL quantities were transferred into 5 mm NMR tubes.

### 4.5. NMR Spectroscopy

NMR spectra were acquired on a Bruker AVANCE III spectrometer operating at 500.13 MHz for ^1^H, at 298 K. The standard 1D spectra were acquired using the “noesypr1d” and “zg” pulse sequences (Bruker library, Rheinstetten, Germany), for aqueous and lipophilic extracts, respectively, with 2.34 s acquisition time, 2 s relaxation delay, 512 scans, 7002.801 Hz spectral width, and 32 k data points. Each free-induction decay was zero-filled to 64 k points and multiplied by a 0.3 Hz exponential function before Fourier transformation. Spectra pre-processing included the manual correction of phase and baseline, and the internal calibration of chemical shifts to TSP or TMS for aqueous and lipophilic extracts, respectively. Then, 2D NMR homonuclear total correlation (TOCSY) and heteronuclear single-quantum correlation (HSQC) spectra were acquired for selected samples to aid spectral assignment, which was supported by comparison with the existing literature and data available on databases, such as Bruker BIOREFCODE (spectral database of AMIX-viewer 3.9.14, Bruker Biospin, Rheinstetten, Germany), human metabolome database (HMDB) [92] and Chenomx NMR Suite (Chenomx Inc, Edmonton, AB, Canada).

### 4.6. Data Processing and Statistical Analysis

The 1D NMR spectra were converted into matrices (AMIX-viewer 3.9.14, Bruker Biospin, Rheinstetten, Germany) after the exclusion of the water (δ 4.6–5.2) and methanol (singlet at δ 3.36) regions for aqueous extracts, and of chloroform and corresponding satellite peaks (δ 7.0–7.5) for lipophilic extracts. Spectra were aligned by recursive segment-wise peak alignment (RSPA) to minimize chemical shift variations (Matlab 8.3.0, The MathWorks Inc., Natick, Massachusetts, USA), and normalized to the total spectral area to reduce the influence of sample concentration. Multivariate analysis was carried out using both unsupervised and supervised methods, namely, principal component analysis (PCA) and partial least squares–discriminant analysis (PLS-DA) upon unit variance (UV) scaling, attributing a comparable weight to each data value (SIMCA-P 11.5; Umetrics, Umeå, Sweden). PLS-DA models were considered statistically robust when corresponding to predictive power (Q^2^) values ≥0.05. PLS-DA loadings were back-transformed, multiplying each variable by its standard deviation, and colored according to variable importance to the projection (VIP) (Matlab 8.3.0, The MathWorks Inc., Natick, MA, USA). The resonances relevant for class separation, identified from PLS-DA loading plots, were integrated (Amix-multi integrate tool 3.9.14, Bruker BioSpin, Rheinstetten, Germany), normalized, and their variations assessed by univariate analysis, combining the calculation of effect size (ES) [74] and statistical significance (Shapiro–Wilk test to assess data normality, Student’s *t*-test or Wilcoxon test for normally distributed or non-normally distributed data, respectively) (R-statistical software). For multiple testing, *p*-values of significantly changed metabolite levels (|ES| > ES error and *p* < 0.05) were corrected by false discovery rate (FDR), based on the Benjamini and Hochberg method [75]. Significant deviations were putatively interpreted based on information derived from the Kyoto Encyclopedia of Genes and Genomes (KEGG) database [93].

## 5. Conclusions

Given the above-described metabolic characteristics for Pd_2_Spm- and cDDP-treated TNBC tumors, a generally stronger impact of the former on polar tumor metabolome is noted, as viewed by NMR, although both metabolic signatures involve the same set of metabolites, which might suggest some similarity regarding the modes of action of both complexes. However, the different magnitudes/directions of polar metabolites’ variations and/or their statistical relevance reveal distinctive patterns, particularly involving alanine/asparagine/glycine metabolic pathways, as well as nucleotides, methylamine and membrane metabolisms. No changes were observed in the lipophilic metabolomes of the tumors. The relationship between the differences in polar metabolomes and the clinical efficacy of Pd_2_Spm compared to cDDP (including hepatotoxicity and cardiotoxicity, although these are expected to be lower for Pd_2_Spm, based on pharmacokinetics) remains unclear at this stage, requiring additional pharmacodynamics and biochemical data from the xenograft model, in order for an unambiguous relationship to be established. 

## Figures and Tables

**Table 1:** Univariate analysis of the polar metabolome of tumors from the MDA-MD-231 CDX mouse model exposed to cDDP and Pd_2_Spm, with comparisons shown (i) for each drug vs. controls, and (ii) between drugs. The metabolite variations selected exhibit |Effect size (ES)| > Error [74], and *p*-value < 0.05. ^‡^ Partial integration of peak. **^a^** Metabolic variations that remain statistically significant after false discovery rate correction [75]. Metabolite abbreviations: Int., intermediates; NAD^+^, nicotinamide adenine dinucleotide (reduced); Ui, unassigned resonance i; other abbreviations as defined in the captions of Figure 1 and Figure 3. Multiplicity abbreviations: s, singlet; d, doublet; m, multiplet.

			cDDP vs. Ctr				Pd_2_Spm vs. Ctr				Pd_2_Spm vs. cDDP			
	Metabolite	δ/ppm (Multiplicity)	ES	±	Error	*p*-Value	ES	±	Error	*p*-Value	ES	±	Error	*p*-Value
Amino acids metabolism	Ala	1.48 (d)	—	—	—	—	—	—	—	—	1.59	±	1.25	1.39 × 10^−2^
	Asn	2.85 (m)	2.25	±	1.51	4.33 × 10^−3^ **^a^**	4.23	±	2.04	2.53 × 10^−3^ **^a^**	1.53	±	1.24	2.26 × 10^−2^
	Gly	3.55 (s)	—	—	—	—	—	—	—	—	–1.32	±	1.2	3.33 × 10^−2^
Membrane precursors	Cho	3.21 (s)	—	—	—	—	2.05	±	1.41	1.39 × 10^−2^	1.81	±	1.29	7.70 × 10^−3^ **^a^**
	PE	3.99 (m)	—	—	—	—	–1.97	±	1.39	3.52 × 10^−2^	–1.46	±	1.23	2.30 × 10^−2^
TCA cycle	Fumarate	6.52 (s)	—	—	—	—	1.6	±	1.31	2.10 × 10^−2^	—	—	—	—
Nucleotides metabolism	ATP	8.54 (s)	2.66	±	1.63	1.78 × 10^−3^ **^a^**	3.02	±	1.67	2.75 × 10^−4^ **^a^**	—	—	—	—
	GA	3.79 (s)	—	—	—	—	–2.09	±	1.42	3.04 × 10^−2^	–1.33	±	1.21	3.25 × 10^−2^
	HX	8.20 (s)	–2.76	±	1.65	1.06 × 10^−2^ **^a^**	–2.61	±	1.55	2.01 × 10^−2^	—	—	—	—
	NAD^+^	8.43 (s)	—	—	—	—	—	—	—	—	1.35	±	1.21	3.93 × 10^−2^
	Uridine	7.86 (d)	—	—	—	—	—	—	—	—	1.27	±	1.19	4.17 × 10^−2^
	UTP	8.00 (d)	2.15	±	1.49	6.80 × 10^−3^ **^a^**	—	—	—	—	1.57	±	1.25	3.69 × 10^−2^
Carbon metabolism	DMA	2.73 (s)	–4.82	±	2.34	1.89 × 10^−4^ **^a^**	-2.39	±	1.49	1.51× 10^−3^ **^a^**	-	-	-	-
Unassigned resonances	U1	4.04 (^‡^)	—	—	—	—	–2.19	±	1.44	3.48 × 10^−2^	–1.40	±	1.22	4.51 × 10^−2^
	U2	8.18 (s)	—	—	—	—	1.56	±	1.31	4.80 × 10^−2^	-	-	-	-
	U3	6.80 (s)	—	—	—	—	—	—	—	—	1.27	±	1.19	3.95 × 10^−2^

## Data Availability

Data available on request due to privacy restrictions.

## Supplementary Materials

The following are available online at https://www.mdpi.com/article/10.3390/ijms221910775/s1, Table S1: List of metabolites and corresponding spin systems identified in the 500 MHz ^1^H NMR spectra of aqueous extracts of TNBC tissues from the MDA-MB-231 cell-derived xenograft (CDX) mouse model; Figure S1: Average 500 MHz ^1^H NMR spectra of lipophilic extracts of tumors from controls group (exposure to vehicle, PBS) of MDA-MB-231 CDX mouse model; Figure S2: Heatmap illustrating the metabolic variations in aqueous extracts of tumors from MDA-MB-231 CDX mouse model relative to the pairwise comparisons cDDP/Pd_2_Spm vs. Controls, and Pd_2_Spm vs. cDDP; Figure S3: Bar chart depicting average intensity ratios of choline compounds.
