# Supplementary material for: Impact of the Pd2Spm (Spermine) Complex on the Metabolism of Triple-Negative Breast Cancer Tumors of a Xenograft Mouse Model
Source: Int J Mol Sci. 2021 Oct 5;22(19):10775. doi: 10.3390/ijms221910775 (PMC8509401; doi:10.3390/ijms221910775)
Supplement: Supplementary file 1 [file ijms-22-10775-s001.zip › ijms-1396154-supplementary.pdf]

International Journal of
Molecular Sciences

MDPI

Supplementary Materials

Impact of the Pd_2Spm(spermine) complex on the metabolism of Triple-Negative Breast Cancer tumors of a xenograft mouse model

Tatiana J. Carneiro [1], Rita Araújo [1], Martin Vojtek [2], Salomé Gonçalves-Monteiro [2], Ana L. M. Bastista de Carvalho [3], Maria Paula M. Marques [3,4], Carmen Diniz [2,*], and Ana M. Gil [1,*]

1 Department of Chemistry and CICECO-Aveiro Institute of Materials, University of Aveiro, 3810-193 Aveiro, Portugal; tatiana.joao@ua.pt (T.J.C.), anarita.asilva@ua.pt (R.A.), agil@ua.pt (A.M.G.)
2 LAQV/REQUIMTE, Department of Drug Sciences, Laboratory of Pharmacology, Faculty of Pharmacy, University of Porto, 4150-755 Porto, Portugal; matovoj@gmail.com (M.V.), salomemonteiro8180@gmail.com (S.G.-M.), cdiniz@ff.up.pt (C.D.)
3 "Química-Física Molecular", University of Coimbra, 3004-535 Coimbra, Portugal; almbc@uc.pt (A.L.M.B.d.C.), pmc@ci.uc.pt (M.P.M.M.)
4 Department of Life Sciences, Faculty of Science and Technology, University of Coimbra, 3000-456 Coimbra, Portugal
* Correspondence: agil@ua.pt (A.M.G.); Tel.: +351 2343700707; and cdiniz@ff.up.pt (C.D.); Tel.: +351 220428608

Content of Supplementary Material:

Table S1. List of metabolites and corresponding spin systems visibly identified in the 500MHz ^{1}H NMR spectra of aqueous extracts of TNBC tissues from the MDA-MB-231 cell-derived xenograft (CDX) mouse model. Arrows represent the qualitative mean abundance of each compound on tumors' tissue comparatively to controls: ↑, increased levels; ↓, decreased levels. Arrows in brackets do not statistical relevance and only indicate a variation tendency. * Possible contamination obtained during the extraction procedure (material disinfection). Metabolite abbreviations: 3-AIBA, 3-aminoisobutyric acid; 3-HBA, 3-hydroxybutyrate; ADP, adenosine diphosphate; AMP, adenosine monophosphate; ATP, adenosine triphosphate; DMA, dimethylamine; GPC, glycerophosphocholine; GSH, glutathione (reduced); IMP, inosine monophosphate; NAD^+, nicotinamide adenine dinucleotide (reduced); PC, phosphocholine; PE, phosphoethanolamine; SAH, *S*-adenosylhomocysteine; TMAO, Trimethylamine *N*-oxide; UDP-GlcA/Glc, uridine diphosphate glucuronate/ glucose; UDP-GlcNAc, uridine diphosphate *N*-acetylglucosamine; UMP, uridine monophosphate; UTP, uridine triphosphate. Multiplicity abbreviations: s, singlet; d, doublet; dd, double doublet; t, triplet; q, quartet; m, multiplet.

Figure S1. Average 500 MHz ^{1}H NMR spectra of lipophilic extracts of tumors from controls group (exposure to vehicle, PBS) of MDA-MB-231 CDX mouse model. * Spectral regions assigned to water (δ 1.48 – δ 1.76), and $CDCl_3$ (and corresponding satellites) (δ 7.00 – δ 7.50), excluded from the multivariate analysis. Abbreviations: Chol., cholesterol; Ester., esterified; FA, fatty acid; PTC, phosphatidylcholine; PTE, phosphatidylethanolamine; PUFA, polyunsaturated fatty acid; SM, sphingomyelin; TG, triglycerides.

Figure S2. Heatmap illustrating the metabolic variations of aqueous extracts of tumors from MDA-MB-231 CDX mouse model relative to the pairwise comparisons cDDP / Pd_2Spm *vs.* Controls (Ctr), and Pd_2Spm *vs.* cDDP. The heatmap is colored according to the Effect Size (ES) in a scale from minimum (blue) to maximum (red) values. ‡ Partial integration of peak. Abbreviations: 3-letter code used for amino acids; ATP, adenosine triphosphate; Cho, choline; Cpd., compound; DMA, dimethylamine; GA, guanidine acetate; HX, hypoxanthine; NAD^+, nicotinamide adenine dinucleotide (reduced); PE, phosphoethanolamine; UTP, uridine triphosphate; Ui, unassigned resonance i.

Figure S3. Bar chart depicting average intensity ratios of choline compounds, obtained with the integration of signals' area for choline, PC and GPC, singlet resonances at 3.21, 3.22 and 3.23 ppm, respectively. Error bars indicate

the respective standard deviation. Asterisk indicates the significance level of 0.05 (* *p*-value < 5E-2). Mice groups are distinguished by the color of each bar: controls, black; cDDP, blue; Pd_2Spm red.

Table S1. List of metabolites and corresponding spin systems visibly identified in the 500MHz ^{1}H NMR spectra of aqueous extracts of TNBC tissues from the MDA-MB-231 cell-derived xenograft (CDX) mouse model. Arrows represent the qualitative mean abundance of each compound on tumors' tissue comparatively to controls: ↑, increased levels; ↓, decreased levels. Arrows in brackets do not statistical relevance and only indicate a variation tendency. * Possible contamination obtained during the extraction procedure (material disinfection). Metabolite abbreviations: 3-AIBA, 3-aminoisobutyric acid; 3-HBA, 3-hydroxybutyrate; ADP, adenosine diphosphate; AMP, adenosine monophosphate; ATP, adenosine triphosphate; DMA, dimethylamine; GPC, glycerophosphocholine; GSH, glutathione (reduced); IMP, inosine monophosphate; NAD$^+$, nicotinamide adenine dinucleotide (reduced); PC, phosphocholine; PE, phosphoethanolamine; SAH, *S*-adenosylhomocysteine; TMAO, Trimethylamine *N*-oxide; UDP-GlcA/ Glc, uridine diphosphate glucuronate/ glucose; UDP-GlcNAc, uridine diphosphate *N*-acetylglucosamine; UMP, uridine monophosphate; UTP, uridine triphosphate. Multiplicity abbreviations: s, singlet; d, doublet; dd, double doublet; t, triplet; q, quartet; m, multiplet.

Metabolite assignment	δ_H ppm (multiplicity, assignment)	HMDB ID [92]	Pd$_2$Spm *vs.* Controls	cDDP *vs.* Controls
**Amino acids and related compounds**				
Alanine	1.48 (d, βCH$_3$); 3.78 (q, αCH)	HMDB0000161	(↑)	(↓)
Asparagine	2.85 (m, βCH$_2$)	HMDB0000168	↑	↑
Aspartate	2.68 (dd, βCH); 2.81 (dd, β'CH); 3.90 (dd, αCH)	HMDB0000191	(↑)	(↓)
Creatine	3.04 (s, N-CH$_3$); 3.93 (s, N-CH$_2$)	HMDB0000064	(↓)	(↑)
Glutamate	2.04 (m, βCH); 2.11 (m, β'CH); 2.36 (m, γCH$_2$); 3.76 (dd, αCH)	HMDB0000148	(↓)	(↓)
Glutamine	2.14 (m, βCH$_2$); 2.45 (m, γCH$_2$); 3.77 (t, αCH)	HMDB0000641	(↓)	(↑)
Glycine	3.55 (s, αCH$_2$)	HMDB0000123	(↓)	(↑)
GSH	2.17 (m, βCH$_2$ Glu); 2.55 (m, γCH$_2$ Glu); 2.96 (m, αCH$_2$ Cys); 3.78 (αCH Glu); 4.57 (m, βCH$_2$ Cys)	HMDB0000125	(↓)	(↓)
Histidine	3.18 (dd, βCH2); 3.99 (dd, αCH); 7.10 (s, C4H ring); 7.98 (s, C2H ring)	HMDB0000177	-	-
Isoleucine	0.94 (t, δCH3); 1.01 (d, β'CH3); 1.99 (m, βCH	HMDB0000177	-	(↓)
Leucine	0.96 (t, δCH$_3$/ δ'CH$_3$); 1.71 (m, βCH$_2$/ γCH)	HMDB0000687	-	-
Lysine	1.45 (m, γCH$_2$); 1.73 (m, δCH$_2$); 1.92 (m, βCH$_2$)	HMDB0000182	(↑)	-
Phenylalanine	7.33 (d, C2H/ C6H ring); 7.38 (m, C4H ring); 7.42 (t, C3H/ C5H ring)	HMDB0000159	(↑)	(↑)
Phosphocreatine	3.05 (s, N-CH$_3$); 3.95 (s, N-CH$_2$)	HMDB0001511	(↑)	(↑)
Proline	3.34 (m, C2H ring)	HMDB0000162		
Taurine	3.27 (t, S-CH$_2$); 3.43 (t, N-CH$_2$)	HMDB0000251	(↓)	-
Threonine	1.33 (d, γCH$_3$); 3.59 (d, αCH); 4.24 (m, βCH)	HMDB0000167		
Tyrosine	6.90 (d, C3H/ C5H ring); 7.20 (d, C2H/ C6H ring)	HMDB0000158	-	(↑)
Valine	0.99 (d, γCH$_3$); 1.05 (d, γ'CH$_3$); 2.27 (m, βCH); 3.61 (d, αCH)	HMDB0000883	(↑)	(↓)
**Choline compounds**				
Choline	3.21 (s, N(CH$_3$)$_3$)	HMDB0000097	↑	(↑)
GPC	3.23 (s, N(CH$_3$)$_3$); 3.92 (m, αCH$_2$); 4.33 (m, PO$_3$-αCH$_2$)	HMDB0000086	(↑)	(↓)
PC	3.22 (s, N(CH$_3$)$_3$); 4.17 (m, PO$_3$-CH$_2$)	HMDB0001565	(↑)	(↑)
**Sugars**				
α-Glucose	3.53 (dd, C2H); 3.83 (m, C4H); 5.23 (d, C1H)	HMDB0003345	-	(↑)
β-Glucose	3.49 (t, C3H); 3.71 (dd, C6H'); 4.65 (d, C1H)	HMDB0003345	-	(↑)

(continued)

**Nucleotides and related compounds**				
Adenosine	4.29 (q, C4′H ribose); 4.44 (dd, C3′H ribose); 6.10 (d, C1′H ribose); 8.12 (s, C8H ring); 8.27 (s, C2H ring)	HMDB0000050	(↓)	(↓)
ADP	6.15 (d, C1′H ribose); 8.27 (s, C2H ring); 8.54 (s, C8H ring)	HMDB0001341	(↑)	(↑)
AMP	4.51 (dd, C2′H ribose); 6.14 (d, C1′H ribose); 8.27 (s, C2H ring); 8.60 (s, C8H ring)	HMDB0000045	(↓)	(↓)
ATP	6.15 (d, C1′H ribose); 8.28 (s, C2H ring); 8.54 (s C8H ring)	HMDB0000538	↑	↑
Hypoxanthine	8.18 (s, C2H); 8.20 (s, C8H)	HMDB0000157	↓	↓
IMP	8.24 (s, C8H ring); 8.58 (s, C8H ring)	HMDB0000175	(↓)	(↓)
Inosine	6.10 (d, C1′H); 8.24 (s, C8H ring); 8.35 (s, C2H ring)	HMDB0000195	(↑)	(↑)
NAD^+	4.50 (m, A3′); 4.54 (m, N2′); 6.04 (d, N1′); 8.18 (s, A2); 8.19 (N5); 8.43 (s, A8); 8.83 (d, N4); 9.15 (d, N6); 9.34 (s, N2)	HMDB0000902	(↑)	-
SAH †	6.08 (d, N-CH-O)	HMDB0000939	(↓)	(↓)
UDP-GlcA/ Glc	5.61 (dd, C1H Glc); 7.95 (d, C2H uridine)	HMDB0000935	(↓)	(↓)
UDP-GlcNAc	5.52 (dd, C1H glucose); 5.95 (d, C5H uridine),	HMDB0000290	(↓)	-
UMP	5.99 (m, C6H ring); 8.11 (d, C5H ring)	HMDB0000288	(↓)	(↓)
Uridine	5.90 (d, C5H ring); 5.92 (d, C1′H ring); 7.88 (d, C6H ring)	HMDB0000285	(↑)	(↓)
UTP	5.97 (m, C5H ring); 8.00 (d, C6H ring)	HMDB0000285	(↑)	↑
**Organic acids**				
3-AIBA †	1.18 (d, αCH)	HMDB0003911	-	(↑)
3-HBA	1.20 (d, CH_3); 2.31 (m, CH_2)	HMDB0000357	(↓)	(↑)
Acetate	1.92 (s, CH_3)	HMDB0000042	(↓)	(↓)
Formate	8.46 (s, CH)	HMDB0000142	(↑)	(↑)
Fumarate	6.52 (s, CH)	HMDB0000134	↑	(↑)
GA	3.79 (s, CH_2)	HMDB0000128	↓	(↓)
Lactate	1.33 (d, CH_3); 4.10 (q, CH)	HMDB0000190	(↓)	(↓)
Succinate	2.41 (s, CH_2)	HMDB0000254	-	(↓)
**Other compounds**				
Acetone	2.24 (s, CH_3)	HMDB0001659	(↓)	-
DMA	2.73 (s, $(CH_3)_2$)	HMDB0000087	↓	↓
Ethanol *	1.19 (t, CH_3); 3.65 (q, CH_2)	HMDB0000108	(↓)	(↑)
m-Inositol	3.28 (t, C5H); 3.62 (t, C4H/ C6H); 4.06 (t, C2H)	HMDB0000211	(↓)	(↓)
PE †	3.99 (m, PO_3-CH_2)	HMDB0000224	↓	(↓)
TMAO	3.27 (s, CH_3)	HMDB0000925	(↓)	-

Figure S1

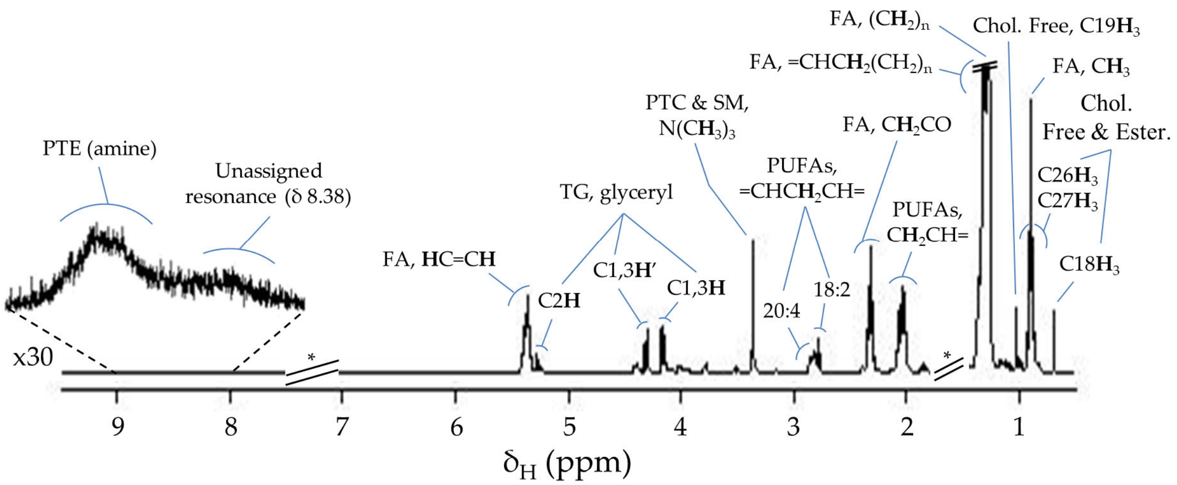

Figure S1. Average 500 MHz ^{1}H NMR spectra of lipophilic extracts of tumors from controls group (exposure to vehicle, PBS) of MDA-MB-231 CDX mouse model. * Spectral regions assigned to water (δ 1.48 – δ 1.76), and $CDCl_3$ (and corresponding satellites) (δ 7.00 – δ 7.50), excluded from the multivariate analysis. Abbreviations: Chol., cholesterol; Ester., esterified; FA, fatty acid; PTC, phosphatidylcholine; PTE, phosphatidylethanolamine; PUFA, polyunsaturated fatty acid; SM, sphingomyelin; TG, triglycerides.

Figure S2

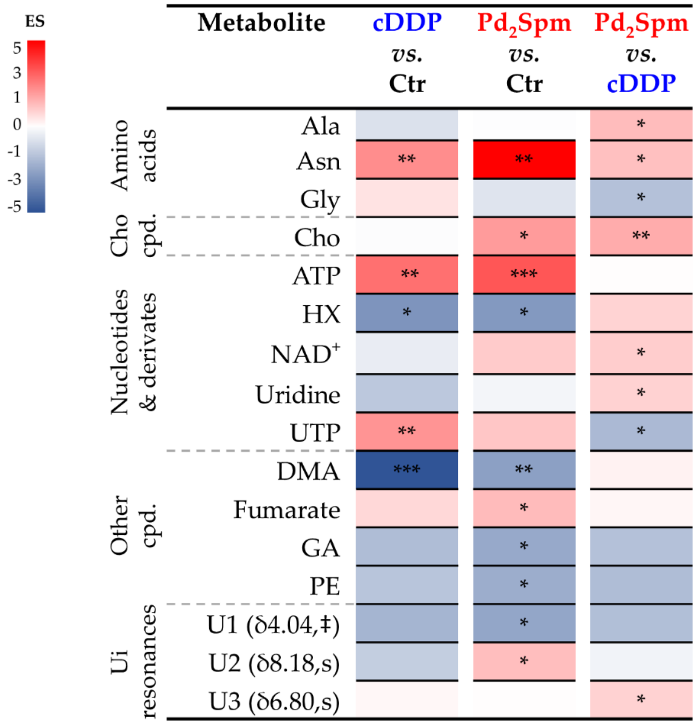

Figure S2. Heatmap illustrating the metabolic variations of aqueous extracts of tumors from MDA-MB-231 CDX mouse model relative to the pairwise comparisons cDDP / Pd_2Spm *vs.* Controls (Ctr), and Pd_2Spm *vs.* cDDP. The heatmap is colored according to the Effect Size (ES) in a scale from minimum (blue) to maximum (red) values. ‡ Partial integration of peak. Abbreviations: 3-letter code used for amino acids; ATP, adenosine triphosphate; Cho, choline; Cpd., compound; DMA, dimethylamine; GA, guanidine acetate; HX, hypoxanthine; NAD^+, nicotinamide adenine dinucleotide (reduced); PE, phosphoethanolamine; UTP, uridine triphosphate; Ui, unassigned resonance i.

Figure S3

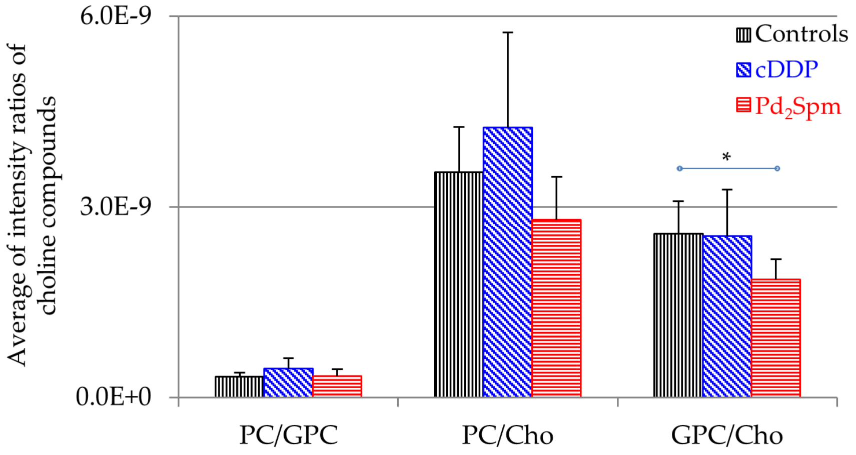

Figure S3. Bar chart depicting average intensity ratios of choline compounds, obtained with the integration of signals' area for choline, PC and GPC, singlet resonances at 3.21, 3.22 and 3.23 ppm, respectively. Error bars indicate the respective standard deviation. Asterisk indicates the significance level of 0.05 (* *p*-value < 5E-2). Mice groups are distinguished by the color of each bar: controls, black; cDDP, blue; Pd_2Spm red.
